# The influence of socio-demographic factors on the stage at which women’s breast cancer is diagnosed and treatment prescribed in the Gaza Strip, occupied Palestinian territory

**DOI:** 10.3332/ecancer.2023.1522

**Published:** 2023-03-20

**Authors:** Walaa Ammar-Shehada, Piet Bracke

**Affiliations:** Health and Demographic Research, Ghent University, 9000 Ghent, Belgium

**Keywords:** breast cancer, Gaza, Palestine, refugees, social determinants of health, health inequities

## Abstract

**Background:**

One of every three women diagnosed with breast cancer (BC) in Gaza does not live for more than 5 years. They are faced by unreliable treatment plans. Radiotherapy is not available locally and there are chronic shortages in the chemotherapy medications. This paper aims to provide understanding of how socio-demographic factors affect the stage at which the cancer is diagnosed and what treatment is prescribed.

**Methods:**

Data were collected through a cross-sectional survey targeting women living in Gaza who had been diagnosed with BC at least once. The survey was self-administered and distributed to 350 women between 1 March 2021 and 30 May 2021. Multinomial logistic regression (SPSS, version 28.0) was used to explore the association between stage of the cancer at diagnosis and socio-demographic characteristics. The relationship between the stage at diagnosis and prescribed treatment was explored using a cluster analysis and crosstabulations.

**Findings:**

Socio-demographic inequalities were reflected in stage at diagnosis and varied by age, education, employment, marital status, and refugee status. Breast cancer was less likely to be detected at an advanced stage among educated respondents (women with primary education OR = 0.093, *p *= 0.008 and women with preparatory education OR = 0.172, *p* = 0.005), employed women (OR = 0.056, *p* = 0.022). It was more likely to be detected at an early stage (OR = 3.954, *p* = 0.011) in women aged 41–50. In widowed and separated/divorced women, it was less likely to be detected at an early stage (OR = 0.217, *p *= 0.029) and (OR = 0.294, *p* = 0.028) respectively, than among married women. Among refugee women, it was less likely to be detected at early stage than among non-refugee women (OR = 0.251, *p* = 0.007). Among the total respondents, only 30% of the full prescribed treatment was available locally.

**Conclusion:**

Our research showed various levels of inequalities at the stage of diagnosis by age, marital status, education, employment and refugee status. Most of the survivors needed treatment that was unavailable locally.

## Background

The protracted emergency in occupied Palestinian territory (oPt) is affecting the health of Palestinians. oPt comprises the West Bank (WB) (including East Jerusalem) and the Gaza Strip (GS). During the 1948 Arab–Israeli war, about 750,000 Palestinians were displaced from their homes and ended up in neighbouring countries such as Jordan, Syria and Lebanon, as well as the parts of Mandate Palestine that became the WB and the GS [1]. The 1948 Palestinian–Israeli war set the stage for decades of healthcare crisis in the territory, with Gaza being the most affected. Within these areas, the combined Palestinian population of around 5.16 million [2] relies on a health system weakened by decades of conflict and instability. With the always hovering prospect of Israeli escalation, the oPt has never made the transition to a post-conflict context; thus, there has been no opportunity for the Palestinians and their healthcare system to experience a recovery phase to ‘recharge’.

Conflict affects the health-seeking behaviour of individuals at various levels because they are forced to choose between fulfilling their basic needs and attending health services, and the lack of health provision in conflict settings [3]. In the GS, this situation is compounded by extreme poverty (53%) [4] and high unemployment rates (47%) [5]. In addition, the health system was recently savaged by the COVID-19 crisis. In April 2020, the number of permit applications for patients and their companions to travel from the GS and WB for treatment abroad declined by more than 90%, compared to the monthly applications in January and February, and referral of GS patients by the Ministry of Health had reduced by 60% [6].

Cancer patients in Gaza face many challenges, such as late diagnosis and lack of standard treatment, which decreases their chances of survival [7]. Breast cancer (BC) incidence was estimated to be 33 per 100,000 in 2018 [8] and the 5-year survival for women with BC (WwBC) diagnosed from 2005 until 2014 was 65.1% [9].

Part of the problem is the late presentation by women with symptoms or late diagnosis by the local health services [10]. With regard to early detection, the Palestinian Ministry of Health recommends that women aged 40–50 years should be screened every 2 years and those who are over the age of 50 should be screened every year [11]. The health system does not send personalised invitations to women, but women who are aware of these recommendations can reach out to governmental facilities for screening free of charge. These recommendations are not evidence-based, and only 2% of women among the target group for mammographic screening had a mammogram in 2021 [12].

Another part of the problem is the unavailability of standard treatment locally; Radiotherapy is not available at all and there are frequent interruptions in chemotherapy. This requires patients to seek treatment outside the GS. However, obtaining a travel permit is a chaotic process which often delays patients’ access to hospitals and puts extra financial and mental pressure on them [13]. The permit approval rate of referred Gazan patients in 2021 was 63% [14]. Of those who died while waiting to receive security permits for travel to destination hospitals, 85% had been referred for cancer treatment and investigation [15]. Furthermore, the low quality of services has had negative effects on oncology patients, particularly women. Palliative care is limited and there is no counselling services for patients and their families.

In the context of the currently fragmented health system and limited financial resources, it is a challenge to offer comprehensive care. Reliable and comprehensive cancer data that are essential for research and policy changes are not easily accessible and obtainable in the GS [8]. Reliable registries and information systems would better identify gaps and help to design interventions. Up to now, no study has been published on the overall socio-demographic characteristics of WwBC in GS.

This paper aims to give an overview of the socio-demographic characteristics of BC survivors in the GS, it also analyses the influence of socio-democratic factors on the stage at which women’s BC is diagnosed and treatment prescribed as well as related inequalities and implications within the broader socio-political context. This understanding will help to guide policies and interventions, especially those that are linked to the health system and social determinants of health.

## Method

### Sample

Data for this paper were collected through a self-administered cross-sectional survey that targeted WwBC living in GS. The survey was distributed to 350 women between 1 March 2021 and 30 May 2021. The estimated number of WwBC in GS (the research population) was 2,058 women. With a confidence level of 95%, the recommended sample size was 324. The selection criteria were: (1) females diagnosed with BC at least once, (2) living in GS

during illness and treatment and (3) still alive. The 350 women were all reachable through the lists of non-governmental organisations (NGOs). Four NGOs cooperated in recruiting cancer survivors registered in their database. The NGOs were selected on the basis of geographical distribution to include survivors from all governorates. The survey was electronic and was shared through closed social media groups (mailing lists, WhatsApp and SMS). The survey acquired data in five sections; socio-demographic, illness history, treatment history and place, types and sources of support received during treatment and changes in survivors’ lives after illness. Data on support and changes in survivors’ lives will be analysed in a separate paper. The response rate was 71%, 251 responses (out of *N* = 350); 1 was excluded and 250 were valid and included in the analysis.

### Dependent variables

*Stage at diagnosis:* According to the Surveillance, Epidemiology and End Results summary stage system, which is the most basic system and is applicable to all anatomic sites, the BC *in situ* stage refers to the presence of abnormal cells that are confined to the layer of cells where they originated (stage 0 or I). *Local stage refers* to invasive cancer that is confined to the breast (stage II). *Regional stage* refers to cancer that has spread to the surrounding tissue and/or nearby lymph nodes (stage III). *Distant stage* refers to cancer that has spread to distant organs and/or lymph nodes, including nodes above the collarbone (stage IV) [16].

*Treatment protocol:* According to Mayo Clinic [17] and breastcancer.org [18], there are different treatment options for BC. Doctors propose protocols based on the type of cancer, its stage, grade, size and whether the cancer cells are sensitive to hormones. Although different pathways could be proposed in cancer treatment, the most common pathway usually starts with *surgery*. If *chemotherapy* is going to be part of care, it is often given as the second. *Radiation* therapy usually follows surgery and chemotherapy. *Hormonal* therapy is often started after other treatments have been given if the cancer is hormone receptor positive.

### Independent variables

*Socio-demographic* information such as: governorate, age, marital status, employment, education, refugee status and place of residence.

*Governorates*: The GS comprises five governorates: North, Gaza, Middle, Khan Yunus and Rafah.

*Age distribution*: The survey was open to women who met the aforementioned criteria; however, the respondents’ age was between 26 and +65 years, and about a quarter of the sample were between 51 and 55 years old. Age is the second-biggest BC risk factor after gender. The incidence rate of BC increases significantly with age and reaches its peak in the age of menopause. None of the survey respondents was below 26 years old, which is not surprising as BC is rare in women younger than 25 years. Age groups were divided into four groups: 26–40, 41–50, 51–65 and >65, keeping in mind the periods of high fertility and menopause.

*Marital status*: Married, single, widowed, divorced and separated were the options that were made available to respondents. Later in the analysis, separated and divorced were merged into one group. The divorced women had formalised everything through the court and were divorced legally, while separated ones were still married on paper but in reality, were no longer married.

*Employment comprised three categories*: Employed women, non-employed and volunteers. The volunteers group comprised working women, but the money paid to them barely covered their transportation and other basic expenses. Thus, they had fewer financial resources than employed women.

*Education* system in oPt encompasses primary, preparatory and secondary levels followed by a higher university degree. Primary education is until the 6th grade, preparatory until the 9th grade and secondary until the 12th grade.

*Palestinian refugees* were defined in 1952 as: ‘persons whose normal place of residence was Palestine during the period from 1 June 1946 to 15 May 1948, and who lost both home and means of livelihood as a result of the 1948 conflict’ [19]. Thus, Palestinian refugees do not fit into the global mainstream definition of refugees who flee their country; in the case of GS and WB, they are more of internally displaced people. Given the specificity of the clause, the UN General Assembly established and mandated the United Nations Relief and Works Agency for Palestine Refugees in the Near East (UNRWA) [20] to serve ‘Palestine refugees’ in five operational areas: Jordan, Lebanon, Syria, GS and WB including East Jerusalem. UNRWA does not administer refugee camps; its responsibility is limited to running education, health, relief and social services.

*History* of *illness and treatment* included frequency of BC diagnosis, stage at diagnosis, number of years with BC, number of treatment years, prescribed treatment, health status at time of completing the survey, place of treatment, types of health facilities accessed and relevant financial factors.

### Data analysis

*Stage at diagnosis*: In order to simplify the analysis, stages I and II were combined as both stages are considered to be early-stage detection, and stages III and IV were combined in a group of advanced-stage detection.

*Treatment prescribed*: In our survey, the responders identified the treatment that had been proposed to them by health providers: surgical, chemotherapy, radiotherapy, hormonal or a combined protocol.

SPSS (version 28.0) was used to perform statistical analysis. *Multinomial logistic regression* was used to determine the relationship between the first dependent variable (BC stage at diagnosis) and the set of independent socio-demographic variables. *Classification and cluster analysis* was used to examine the similarities in the combination of treatment protocols. Four clusters resulted from the cluster analysis: 39.6% (99/250) of the cases in cluster #1 had been prescribed both chemotherapy and surgical treatment, 47.6% (119/250) of the cases in cluster #2 had been prescribed all kinds of treatment, 3.6% (9/250) of the cases in cluster #3 had been prescribed radiotherapy and chemotherapy and 9.2% (23/250) of the cases in cluster #4 had been prescribed hormonal and surgical treatment. Finally, crosstabs was used to describe the association between clustered cases and the (stage at diagnosis) variable, which turned out to be significant (*p* value = 0.007).

### Ethical approval

The Helsinki Committee for Ethical Approval of the Palestinian Health Research Council provided written approval for carrying out this research (approval PHRC/HC/730/20). The researcher was also given permission to collect data from the oncology departments of Ministry of Health facilities. Because of COVID-19 movement restriction and physical distancing measures that were imposed at the time of data collection, an electronic survey was used to avoid delay. A communication channel with women’s contact information had to be found to reach women remotely. The administrations of four NGOs that were working with WwBC approved and supported the distribution of survey forms through their lists and social media groups. Construct, content and face validity of the survey were checked by three public health experts and their comments were considered in the survey prior its piloting with ten women. After the survey was finalised, the online link was shared with the administration of NGOs for dissemination among BC survivors. The survey was in Arabic, the local language in Palestine. The first page included an introduction to the survey, its purpose and the main eligibility criteria, followed by a consent statement. If a woman agreed to fill out the form and checked the consent box, she was forwarded to the next section of the survey. The two authors had access to the collected data. Confidentiality and anonymity were safeguarded while collecting, storing and analysing data.

## Results

### Population socio-demographic characteristics

Table 1 displays the number and percentage of variables related to socio-demographic, illness and treatment factors. Of the 250 women who responded to the survey, 34.8% (87/250) were from Gaza, 20.4% (51/250) from Middle, 18.4% (46/250) from Rafah, 15.2% (38/250) from Khan Yunus and 11.2% (28/250) from North governorate. The distribution of the population in 2017 in the five governorates was 19.4% in North, 34.4% in Gaza, 14.4% in Middle, 19.5% in Khan Yunus and 12% in Rafah [21]. When the geographical distribution of respondents is compared with the general distribution of the population in GS, it is clear that the sample was geographically representative. The average age of respondents was 48.4 years and almost three-fourths of the respondents (182/250) were married. About two-thirds (156/250) of the women reported secondary school educational level. 88.8% (222/250) were non-working and 11.2% (28/250) had some sort of income through employment (16/250) and voluntary work (12/250). The number of employed women among non-refugee respondents was 14% (17/172), which was higher than the 10% (11/78) among refugee respondents. Slightly more than two-thirds of respondents 68.8% (172/250) were refugees and 31.2% (78/250) were non-refugees whose family origin was from the GS. 66% (165/250) of women lived in cities while only 34% (85/250) lived in camps. There are more than four generations of refugees in GS and factors such as work, the interrelation between city and camp life, as well as availability of services make living in cities a better option for the refugees who have the opportunity to do so.

### Personal practices and use of health services

*Detection:* The average number of total illness years was 6 years (SD = ±4.59) and the average number of treatment years was 5.4 years (SD = ±4.19) (see Appendix). About three-fourths 72% (180/250) of respondents had been diagnosed with BC once (Table 1). 29.2% (73/250) had stage I, 32.8% (82/250) had stage II, 15.6% (39/250) had stage III and 2.8% (7/250) had stage IV at diagnosis. 19.6% (49/250) did not know their stage at diagnosis (Table 2). It is notable that about one-fifth of the sample did not know their BC stage. Further analysis showed that 82% (40/49) of the women who did not know their stage at diagnosis were refugees. Currently, there is no comprehensive health information system in place through which women can digitally access their health files and know their status. Women can obtain paper reports to access further procedures, and patients themselves administer their files among different health care providers. Only 29.6% (74/250) of survey respondents had their prescribed treatment in the GS, while 65.6% (164/250) had to receive their treatment both in and outside the GS, while 4.8% (12/250) had their treatment outside the GS (Table 1).

Table 3 summarises the results of multinomial logistic regression analysis in both groups (early and advanced-stage detection) using “women who did not know” as a reference category. BC is less likely to be detected in an advanced stage in educated women (Women with primary education OR = 0.093, *p* = 0.008 and women with preparatory education OR = 0.172, *p* = 0.005). This also holds for BC in employed women (OR = 0.056, *p* = 0.022). Controlling for age, the same groups remained significant, with BC being less likely to be detected at an advanced stage in primary/preparatory educated (OR = 0.097, *p* = 0.008), secondary educated (OR = 0.177, *p* = 0. 005) and employed (OR = 0.048, *p* = 0.015) women.

Women aged 41–50 years were a significant group among all age groups to that are more likely to have BC detected at an early stage (OR = 3.954, *p* = 0.011). It is less likely to be detected at an early stage in widowed and separated women (OR = 0.217, *p* = 0.029) and (OR = 0.294, *p* = 0.028) respectively, compared with married women. It is less likely to be detected at an early stage (OR = 0.196, *p* = 0.037) in volunteers. Refugee women are less likely to be diagnosed at an early stage in comparison with non-refugee women (OR = 0.251, *p* = 0.007). Even when the early-detection group is controlled for age, BC continues to be less likely to detected at an early-stage among refugee women (OR = 0.339, *p* = 0.024). This also applies to widowed women (OR = 0.203, *p* = 0.020).

### Prescribed treatment

Table 2 shows that 95.6% (239/250) of the sample respondents underwent surgical procedures. Of those, 71.1% (170/239) had radical bilateral or unilateral mastectomy, and 16.7% (40/239) had partial bilateral or unilateral mastectomy. 12.1% (29/239) had lumpectomy.

In the hierarchical cluster analysis summarised in Table 4, slightly less than half of the cases (47.6%) were observed to be in cluster 2 where a combination of four treatment protocols was prescribed: surgical, chemotherapy, radiotherapy and hormonal. That was followed by 39.6% of cases in cluster 1 who were prescribed a combination of surgical and chemotherapy protocols. Cluster 2 protocols were used to treat over half of the women diagnosed at both early and advanced stages and slightly over a quarter of women who did not know their stage. Cluster 1 protocols were used to treat a little less than half of the women in the advanced-stage and less than half of those who did not know their stage, compared with about a third of women in the early-stage.

## Discussion

Our study aimed to provide understanding of socio-demographic variation among WwBC in stage at diagnosis. WwBC in Gaza are not all the same and they face various levels of inequality according to their socio-economic characteristics. We found that inequalities abound not only in stages at which BC is diagnosed, but also in accessing prescribed treatment. The inequalities in access for the group as a whole are due to non-neutralisation of the right to health from the broader socio-political dynamics, including treatment not being available locally and failure to ensure unrestricted access to essential services on time. The violation of right to health affects women differently because their socio-economic status is different, and the disadvantaged are affected more.

### Socio-demographics and detection

This study has shown that BC is less likely to be diagnosed at an early stage in women having characteristics such as being widowed, separated/divorced and refugee status. The association of marital status with diagnosis has been studied in other countries. Osborne *et al* [22] concluded that unmarried women are more likely to be diagnosed at BC II–IV rather than stage I, and that this could be due to the benefits that enjoyed by married women is derived from intrinsic social support and social networks available to them. Another study by Montazeri *et al* [23] found that widowed and divorced women have higher risk of delay than married women do. The study found that widowed and divorced women do not have enough motivation to seek help or care about themselves and that they lack support. Refugee women within Palestine, too, have greater vulnerabilities and health needs. With regard to predisposing characteristics, and after controlling for age, BC was detected at an early stage among fewer refugee women. Among respondents who did not know their stage at diagnosis, four-fifths were refugees, which is a reflection of their insufficient access to information. This group of women faces additional barriers to accessing health care, depending on the degree of social protection available to them [24]. They generally have less access to resources. As observed earlier in the paper, employment is 40% higher among non-refugees than that among refugee women. The family financial resources of refugees are limited, and the services offered to second, third and fourth generations of refugees are much fewer especially with the financial crises that have occurred in funding UNRWA [25] services in recent years. The eight refugee camps in the GS are adjacent to cities, but they are of temporary structures and overcrowded. UNRWA is not mandated to maintain camps, that is left to the host countries. Palestine refugees living in Gaza, especially those living in camps, are at the bottom of the socio-economic ladder [26]. Although UNRWA supports efforts to raise awareness about BC in the community, it is not among the providers of services relating to BC treatment. When it comes to cancer care, refugees tend to face more barriers in accessing services throughout the Middle East because of their poor living conditions and limited resources [27].

Although there are 17 mammography machines in GS, only 5 are owned by the Ministry of Health and offer women free imaging [28, 29]. At the time of this study, one of those was not functioning. Of the other four, three were used for diagnostic services and one for screening [29]. The situation in GS shows that when a population lives in poverty and faces barriers to access, including affordability, making primary health services available does not mean they will be used. Women are constantly negotiating to secure daily necessities for their families and may not also be able to pay for transport to go to a health facility. Thus, disadvantaged women such as those without jobs and refugees have unequal access to services, which could later lead to a bad prognosis.

There is no organised screening programme in Gaza; therefore, socio-cultural factors and women’s knowledge and attitudes are particularly influential in determining their health practices [28]. In a study by Lafi and Shaheen [30], although four-fifths of surveyed women were positive about seeking healthcare when they needed it, only a quarter practised breast self-examination, and 87% had never had a mammogram or been offered a breast examination. Ashour [31] concluded that such behavioural changes among Gazan women are heavily influenced by multiple factors: ability to pay for care, the household’s health conditions and entitlement to certain services. These factors determine women’s choice and influence healthcare-seeking behaviour. Gazan households use a wide range of coping mechanisms to minimise the use of health services, including self-medication and decreasing use of the private sector. Gazan women use health services when they have serious health problems; hence, they go for mammography when they are referred by a doctor for diagnostic purposes rather than through a screening invitation. Some efforts are being made to increase community awareness [32, 33]; for example, a campaign is organised during the month of October each year in Gaza to encourage women to learn how to do self-examination and to undergo screening. Gaza, with its complex situation and evident barriers to health and as a low-middle income country, needs to prioritise early detection strategies. More targeted and systematic initiatives could help to increase the percentage of women diagnosed at early stages and eliminate an avoidable delay.

### Post-detection and access to radiotherapy and chemotherapy

The low percentage of women who had prescribed treatment available locally means that fewer than one-third of the survey respondents had the chance to fully access their treatment within the GS. The other women had to travel to receive or complete their treatment protocol. The unavailability of many chemotherapy items and lack of radiotherapy treatment make it necessary for some patients to be referred to facilities outside the GS where they can receive or continue their treatment.

Drug shortages have been a chronic problem for more than two decades. As reported by the Ministry of Health in Gaza, there was less than a month’s stock of almost half (46%) of the medicines in the Essential Medicines List remaining at the time of monthly stock takes in 2018 [34]. In August 2018, after irregular supplies from the Palestinian Authority for 18 months, the Ministry of Health in Gaza announced that chemotherapy would not be available any more, and the shortage of medicines peaked at its highest rate of 75% out of stock [35]. In March 2022, the Ministry of Health in Gaza highlighted the medical shortage again, especially for cancers, where the shortage in drugs was about 50% [36]. Although the chronic shortages of drugs span the oPt, there is a difference between shortages in WB and GS. In 2019, 42% of the medicines in the essential medical list in the GS were completely depleted and there was less than a month’s supply of 26% of essential medical disposables. In contrast, there was 97% overall availability in the WB in 2018 [37]. This gap widened after the political divide between Fatah and Hamas began in 2007, and the supply of medicines through the Ministry of Health in Ramallah became irregular. The supply irregularity created shortages of drugs, which broke the cycle of treatment where the treatment protocol required one or more of unavailable medicines. The lack of a clear timeframe for making these medicines available again could delay treatment plans. This is particularly in the case of women who need chemotherapy medications. All women who need radiation therapy and PET/CT scans have to be referred to institutions outside the GS because, by listing them on the dual-use list, Israel does not allow radiotherapy machines into the GS [38]. According to data from Augusta Victoria Hospital, one of the two main destination hospitals for cancer patients in East Jerusalem, around 80% of BC cases reach the hospital at a palliative stage [28], which shows the substantial delay that women face during the diagnosis and treatment procedure. It adds to the time needed for obtaining security clearance from Israel, after which there is delay in applying for and receiving a permit for travel to the destination hospital. The delays evidently cause higher mortality among patients who are unsuccessful in reaching the hospital and receiving treatment on time [38]. This uncertainty is structural in WwBC treatment plans: Is treatment available within Gaza? if not, will they travel? If yes, will they be allowed to have a companion? There are no certain answers. All this has to be handled by women while bearing their ongoing physical pain.

### Surgical treatment: mastectomy and breast reconstruction (BR)

As seen earlier, 95.6% (239/250) of the women respondents needed surgery as part of their prescribed protocols, including 68% (170/250) of the respondents who underwent radical mastectomies. This is in line with other research in Gaza, where two-thirds of the sample (133/173) had undergone radical mastectomy [39]. A higher proportion of women have mastectomies than those who have lumpectomies [29, 40]. The reasons for this include late detection and confirmed diagnosis, but it has been viewed mainly as resulting from lack of radiotherapy even though there has been global change to lumpectomy for most cases as the standard of care. Despite the high number of women undergoing surgery, only one respondent 0.4% said she had undergone BR surgery, while 98% said no, and 1.6% said they did not know what the term means.

Surgeons are aware of the barriers to accessing radiotherapy treatment outside Gaza and they opt for mastectomy to spare women that extra risk [29, 40]. Women do not object to more radical intervention even though they fear that it would affect their marital relationship and might lead to divorce [39]. They often do not discuss the decision and choose to risk their lives while undergoing other (combination of) treatments that are unlikely to be guaranteed. In a patriarchal community like the GS, married women are keen to maintain the constructed ‘perfect image’ in the eyes of their husbands. They fear divorce or the arrival of a second wife. Generally, Palestinian women living in Gaza are financially dependent on their husbands and if the marriage is threatened after a woman’s illness, she will rarely refuse divorce or a second wife [41]. Eman Shannan, Manager of Aid and Hope Program for Cancer Patients Care in Gaza [42], which provides services to WwBC, confirmed in an e-mail sent to the researcher that 70% of women registered at her organisation had been abused or abandoned because of their illness.

In such a situation, Gazan women are likely to view BR surgery as a priority, not a complementary procedure. Studies in other countries that share many of the social composition features of the Palestinian community, such as social structure, religious and cultural values, emphasise that, as discussing marital problems is a taboo, such problems are being covered up by silence. That is the case with BC survivors after mastectomy in an Iranian society [43]. Another qualitative ethnographic study explores BC in a patriarchal setup and the stigma attached to it in Pakistan: ‘It is seen that breast cancer poses a threat to femininity as it leads to a distortion of beauty ideals and disrupts the domestic roles of women as housewives and mothers, which stigmatizes them, and they have to make multiple efforts to restore themselves physically and mentally’ [44].

No specific studies have been conducted in Palestine on such sensitive topics, but a few researchers have highlighted the issue as part of exploring post-cancer quality of life [45] and as part of the multiple difficulties facing survivors in the WB, such as a socio-cultural context that stigmatises cancer and encourages its concealment [46]. Researchers have also brought out how these socio-cultural attitudes may have unintentionally negatively affected survivors through mainstream norms related to a culture of shame and stigmatisation of illness. Women are expected to accommodate others’ expectations and avoid embarrassment or being pitied. Such beliefs, practices and expectation lead to survivors feeling insecure about their body image, which affects their status as wives [47]. These studies in similar contexts show that body image can be life-changing for women in a society with a complex social and power structure. The definition of good and bad is shaping the dynamics that women experience through their ‘un-healing’ process. The low utilisation of BR is either due to lack of awareness [48] or lack of financial resources [49] in addition to other factors such as insufficient prior knowledge [48]. Therefore, until improvements are made to have more effective screening and detection strategies, post-treatment options such as BR need to be supported so that women face fewer social challenges because of their illness.

## Conclusion

In conclusion, our research showed different levels of inequality through socio-demographic characteristics at the stage of diagnosis, as well as at the treatment stage by availability and accessibility for the whole group of BC survivors in the GS. There are also inequalities in the limited post-treatment options.

In the absence of a national BC screening programme, detecting the disease is left to personal factors and capacity to access resources. Gazan women are not a homogenous group. It is significant that BC is less likely to be detected in widowed, separated/divorced and refugee women at an early stage. Taking age into account as a major risk factor, refugee women are still far from detection at an early stage. However, employment and education are the main characteristics that make it less likely that women will be confronted with the disease at an advanced stage. This shows a social gradient in access to services. Timely access to services determines the later treatment protocols, and early-stage detection of cancer increases successful containment of illness and improves chances of survival. Hence, BC detection seems to be attributed to structural features of the community rather than to the composition of the population. This means that the position of women and the surrounding institutional characteristics of healthcare and social structure in which women have to bargain their health needs for basic livelihood has affected stage of diagnosis. These factors should be considered when aiming to increase access to healthcare.

Unavailability or interruption in making the prescribed treatment available locally in the GS is an additional burden on patients. When patients need to be referred to hospitals outside Gaza for treatment, they face multi-layered barriers such as the travel permit system. The organisation and distribution of services is not only impacted by the Israeli–Palestinian conflict, but also by the internal Palestinian dispute. Lack of equipment such as radiotherapy machines limits treatment options in Gaza. Women undergo more radical surgical procedures to save their lives, but they are confronted by social injustice. They are often seen as ‘awaiting death’, which impacts the quality of their lives. This has a long-lasting impact on the social life of ill women.

Addressing social determinants of health is crucial for minimising health inequalities. Overall driving forces that impact health in all domains should not be ignored when interventions are designed. These forces such as social position, income inequality, institutional discrimination and limited access to resources have been generated by the larger geo-political power structures, which needs to be considered when aiming at minimising health inequalities.

## Recommendation

The study recommends that policy-makers should adopt a more integral approach to improving access to cancer screening by considering socio-demographic factors when designing targeted strategies to advancing equity. Researchers, too, need to keep in mind that as the dynamics surrounding women are different and affect their access to health services differently, a more qualitative perspective is needed to unpack their vulnerabilities, to examine how women make their decisions and how they face social change. This study recommends in-depth qualitative analysis of the conditions surrounding WwBC, especially among refugees, to draw conclusion why they are less represented in the early-detected group. Also, it is necessary to focus future research on formal and informal sources of support for ill women as the conditions surrounding ill women can have harsh effects on health and self-image, all of which affects their social position and hence their quality of life.

## Limitations

Our survey was self-administered and, due to the COVID-19 situation and imposed restrictions, it had to be in an electronic form. That means some women who were technologically illiterate could have been dependent on a family members to fill it in, which could limit the respondent’s privacy.

The fact that this study is targeting BC survivors, as only alive WwBC are able to complete the survey, which means that there might have been a distortion of the sample in such a way that there were fewer advanced-stage respondents.

Due to institutional differences between WB and GS, the study data are not generalisable. It is not possible to study health issues irrespective of the socio-political segregation that affects them. These institutional factors differ from region to region, and the situation in GS is quite unique and incomparable to any other area even within oPt.

External researchers and social scientists usually have limited access to the local context and have limitations in interacting with patients in such an isolated geographical area. This study presents data that has not been researched before but it can still be useful in guiding policy-makers to adopt a more comprehensive approach to minimising health inequalities.

## Authors’ contributions

The first author (Walaa Ammar-Shehada) had the responsibility of study design, conceptualisation, data collection, validation and analysis in addition to writing and reviewing the manuscript.

The co-author (Piet Bracke) critically reviewed the manuscript, advised on the conceptualisation, methodology and presentation of the results and provided overall supervision.

All authors gave final approval for the paper to be published. All authors have seen and approved the final version of the abstract and manuscript for publication.

## Conflicts of interest

The authors declare no conflicts of interest.

## Funding

None.

## Figures and Tables

**Table 1.** Number and percentage of variables related to socio-demographic, illness and treatment factors (*N* = 250).

Variable	Number	%
**Governorate**		
North	28	11.2
Middle	51	20.4
Khan Yunus	38	15.2
Rafah	46	18.4
Gaza	87	34.8
Age		
26–40	68	27.2
41–50	61	24.4
51–65￼	106	42.4
>65	15	6.0
Marital status		
Widow	19	7.6
Single	20	8.0
Married	182	72.8
Separated/divorced	29	11.6
Educational level		
Primary and preparatory	25	10
Secondary education	156	62.4
Bachelor, Masters or PhD	69	27.6
Number of children		
0	41	16.4
1–4	83	33.2
5–11	106	42.4
NA (not previously married)	20	8
Employment		
Other (volunteer)	12	4.8
Employed	16	6.4
Not employed	222	88.8
Refugee/non-refugee		
Refugee	172	68.8
Non-refugee	78	31.2
Residential area		
Camp	85	34.0
City	165	66.0
Number of detection times		
Once	180	72
Twice	55	22
More than two times	15	6.0
Place of treatment		
In the GS	74	29.6
In and outside the GS	164	65.6
Outside the GS	12	4.8
Health status when woman completed the survey		
Under treatment	166	66.4
Recovered	10	4.0
Finished treatment and undergoing checkups	73	29.2
Other	1	0.4

**Table 2.** Stage at diagnosis and treatment prescribed by socio-demographic characteristics (*N* = 250).

		Stage at diagnosis				Treatment prescribed			
		Early stage (I and II)	Advanced stage (III and IV)	Did not know	Total	Surgical	Radiotherapy	Chemotherapy	Hormonal
		155 (62%)	46 (18.4%)	49 (19.6%)	250	239^a^ (95.6%)	162 (64.8%)	213 (85.2%)	169 (67.6%)
Age									
	26–40	40 (58.8%)	16 (23.5%)	12 (17.6%)	68 (100%)	63 (92.6%)	43 (63.2%)	61 (89.7%)	48 (70.6%)
	41–50	49 (80.3%)	3 (4.9%)	9 (14.8%)	61 (100%)	59 (96.7%)	40 (65.6%)	50 (82.0%)	47 (77.0%)
	51–65	58 (54.7%)	22 (20.8%)	26 (24.5%)	106 (100%)	103 (97.2%)	71 (67.6%)	88 (83.8%)	63 (60.0%)
	>65	8 (53.3%)	5 (33.3%)	2 (13.3%)	15 (100%)	14 (93.3%)	8 (53.3%)	14 (93.3%)	11 (73.3%)

Marital status	Widow	7 (36.8%)	7 (36.8%)	5 (26.3%)	19 (100%)	18 (94.7%)	13 (68.4%)	17 (89.5%)	11 (57.9%)
	Single	14 (70.0%)	2 (10.0%)	4 (20.0%)	20 (100%)	17 (85.0%)	8 (40.0%)	18 (90.0%)	14 (70.0%)
	Married	120 (65.9%)	32 (17.6%)	30 (16.5%)	182 (100%)	177 (97.3%)	130 (71.8%)	156 (86.2%)	126 (69.6%)
	Separated/divorced	14 (48.3%)	5 (17.2%)	10 (34.5%)	29 (100.0%)	27 (93.1%)	11 (37.9%)	22 (75.9%)	18 (62.1%)

Employment	Volunteer	5 (41.7%)	2 (16.7%)	5 (41.7%)	12 (100%)	12 (100.0%)	6 (50.0%)	10 (83.3%)	9 (75.0%)
	Working	10 (62.5%)	1 (6.3%)	5 (31.3%)	16 (100%)	14 (87.5%)	8 (50.0%)	13 (81.3%)	7 (43.8%)
	Non-working	140 (63.1%)	43 (19.4%)	39 (17.6%)	222 (100%)	213 (95.9%)	148 (67.0%)	190 (86.0%)	153 (69.2%)

Educational level	Primary/preparatory	13 (52.0%)	4 (16.0%)	8 (32.0%)	25 (100%)	24 (96.0%)	14 (56.0%)	20 (80.0%)	17 (68.0%)
	Secondary	101 (64.7%)	24 (15.4%)	31 (19.9%)	156 (100%)	150 (96.2%)	105 (67.3%)	130 (83.3%)	104 (66.7%)
	Undergraduate/postgraduate	41 (59.4%)	18 (26.1%)	10 (14.5%)	69 (100%)	65 (94.2%)	43 (63.2%)	63 (92.6%)	48 (70.6%)

Refugee/non-refugee	Refugee	98 (57.0%)	34 (19.8%)	40 (23.3%)	172 (100.0%)	163 (94.8%)	108 (62.8%)	147 (85.5%)	121 (70.3%)
	Non-refugee	57 (73.1%)	12 (15.4%)	9 (11.5%)	78 (100.0%)	76 (97.4%)	54 (70.1%)	66 (85.7%)	48 (62.3%)

a 29 (12.1%) lumpectomy, 170 (71.1%) radical bilateral or unilateral mastectomy and 40 (16.7%) partial bilateral or unilateral mastectomy

**Table 3.** Multinomial logistic regression analysis of BC stage at diagnosis by socio-characteristics (with and without controlling for age) (N = 250).

Stage at diagnosis^a^	Early-stage detection (stage I and stage II)								Advanced-stage detection (stage III and stage IV)							
						Adjusted								Adjusted		
	B	Std. error	Sig.	Exp (B)	B	Std. error	Sig.	Exp (B)	B	Std. error	Sig.	Exp (B)	B	Std. error	Sig.	Exp (B)
Governorate																
North	−0.529	0.697	0.448	0.589	−0.438	0.689	0.525	0.645	−0.788	0.821	0.337	0.455	−0.902	0.821	0.272	0.406
Middle	−0.932	0.592	0.116	0.394	−0.554	0.561	0.324	0.575	−0.644	0.719	0.371	0.525	−0.910	0.691	0.188	0.403
Khan Yunus	−1.184	0.581	0.042	0.306	−0.942	0.554	0.089	0.390	−1.814	0.856	0.034	0.163	−2.150	0.848	0.011	0.116
Rafah	−0.418	0.557	0.453	0.658	−0.480	0.550	0.383	0.619	−0.961	0.690	0.164	0.383	−1.202	0.697	0.085	0.301
Gaza	0^b^	.	.	.	0^b^	.	.	.	0^b^	.	.	.	0^b^	.	.	.
Age					−0.082	0.209	0.695	0.921					−0.154	0.251	0.538	0.857
≥65	0.390	0.906	0.667	1.477					0.755	1.001	0.451	2.127				
26–40	−0.006	0.482	0.989	0.994					0.125	0.567	0.825	1.133				
41–50	1.375	0.542	0.011	3.954					−0.906	0.833	0.277	0.404				
51–65	0^b^	.	.	.					0^b^	.	.	.				
Marital status																
Widow	−1.530	0.702	0.029	0.217	−0.1.594	0.686	0.020	0.203	0.154	0.749	0.837	1.166	0.262	0.733	0.721	1.300
Single	−0.096	0.719	0.894	0.909	−0.437	0.683	0.523	0.646	−1.912	1.053	0.069	0.148	−1.761	1.009	0.081	0.172
Separated/divorced	−1.224	0.558	0.028	0.294	−0.936	0.528	0.076	0.392	0.276	0.700	0.694	0.759	−0.430	0.682	0.528	0.651
Married	0^b^	.	.	.	0^b^	0.	.	.	0^b^	.	.	.	0^b^	.	.	.
Employment																
Volunteer	−1.630	0.783	0.037	0.196	−1.386	0.763	0.069	0.250	−1.252	1.019	0.219	0.286	−1.596	1.002	0.111	0.203
Employed	−1.111	0.733	0.130	0.329	−1.069	0.711	0.133	0.343	−2.881	1.263	0.022	0.056	−3.036	1.249	0.015	0.048
Not employed	0^b^	.	.	.	0^b^	.	.	.	0^b^	.	.	.	0^b^	.	.	.
Educational level																
Primary/preparatory	−0.974	0.737	0.186	0.378	−1.113	0.698	0.111	0.329	−2.371	0.894	0.008	0.093	−2.330	0.873	0.008	0.097
Secondary	−0.442	0.538	0.411	0.643	−0.512	0.521	0.326	0.600	−1.759	0.629	0.005	0.172	−1.734	0.616	0.005	0.177
Undergraduate or postgraduate	0^b^	.	.	.	0^b^	.	.	.	0^b^	.	.	.	0^b^	.	.	.
Refugee status																
Refugee	−1.381	0.509	0.007	0.251	−1.081	0.481	0.024	0.339	−0.656	0.629	0.297	0.519	−0.644	0.609	0.290	0.525
Non-refugee	0^b^	.	.	.	0^b^	.	.	.	0^b^	.	.	.	0^b^	.	.	.
Residential area																
Camp	−0.158	0.433	0.716	0.854	−0.240	0.422	0.569	0.787	0.603	0.550	0.273	1.828	0.656	0.543	0.227	1.927
City	0^b^	.	.	.	0^b^	.	.	.	0^b^	.	.	.	0^b^	.	.	.

a The reference category is: `Women who did not know’.

b This parameter is set to zero because it is redundant.

**Table 4.** Cluster analysis of treatment protocols and crosstabulation^a^ of cluster and stage of BC (*N* = 250).

	Clusters			
	1. Chemotherapy and surgical treatment	2. All treatments	3. Radiotherapy and chemotherapy	4. Hormonal and surgical treatment
Stage at diagnosis				
Early-stage	36.1%	52.9%	2.6%	8.4%
Advanced stage	43.5%	52.2%	2.2%	2.2%
Women who did not know	46.9%	26.5%	8.2%	18.4%
Total *N* (%)	99 (39.6%)	119 (47.6%)	9 (3.6%)	23 (9.2%)

a Pearson chi-square = 0.007, Likelihood ratio = 0.007

## Appendix

**Table:** Number of years with illness and treatment (*N* = 250)

Number of years with illness (M ± SD)		6 ± 4.59
	Number	Percentage
≤5 years	144	58
6–10 years	78	31
>10 years	28	11
Total	250	100
Total number of treatment years		5.4 ± 4.19
≤5 years	162	65
6–10 years	69	28
>10 years	19	8
Total	250	100
